# “Just another thing for me to stress off of:” Responses to Unintentional Fentanyl Use in a Community-based Study of People who Use Opioids

**DOI:** 10.21203/rs.3.rs-2842551/v1

**Published:** 2023-04-27

**Authors:** Jennifer Lorvick, Jordana Hemberg, Madeleine George, Joy Piontak, Megan Comfort

**Affiliations:** RTI International; RTI International; RTI International; RTI International; RTI International

**Keywords:** unintentional fentanyl use, fentanyl, overdose, harm reduction

## Abstract

The unintentional consumption of fentanyl is a serious health risk for people who use illicit drugs. In an ongoing community-based study regarding polysubstance use among people who use opioids, we found that 17 of 58 (29%) of participants who did not endorse fentanyl use in the past thirty days tested positive for fentanyl during point-of-care urinalysis (UA). This paper describes the reactions and experiences of participants who were informed they had consumed fentanyl unintentionally, as well as how the research team handled the unanticipated occurrence of discordant results. Consistent with other recent studies, we found that people learning of unintentional fentanyl use expressed strong concerns about accidental overdose. It was common for participants to reflect on recent substance use experiences that were atypical and might have involved fentanyl, as well as to examine sources of recent drug purchases. While not all participants were surprised that they had unintentionally consumed fentanyl, all felt that learning their positive results was important due to risk of overdose. Research and medical staff who routinely conduct urinalysis have an opportunity to promote awareness of possible contamination by sharing and discussing UA test results with people who use drugs in non-judgmental manner. In addition to the widely promoted harm reduction strategy of testing drugs with fentanyl test strips, self-administered UA, particularly after an unexpected reaction to using a drug, could provide useful information for people buying and using illicit drugs.

## Introduction

The growing presence of fentanyl in illicit drug markets has driven a dramatic escalation in opioid overdoses.^1^ According to the Centers for Disease Control, 82% of opioid overdose deaths in 2020 involved illegally manufactured synthetic opioids, namely fentanyl and fentanyl analogues such as carfentanil.^2^ Fentanyl has steadily moved Westward in the U.S. during the past decade,^3^ and, in 2021, was present in 83% of opioid overdose deaths in California.^4^

The term ‘fentanyl-involved’ reflects an important characteristic of illicit fentanyl use, which is that it frequently occurs in the context of polysubstance use. National data from patients entering substance use treatment in 2022 indicate that 95% of people testing positive for heroin and 57% of people testing positive for methamphetamine also tested positive for fentanyl.^5^ Toxicological data in overdose cases clearly show an increasing presence of fentanyl in heroin overdose cases.^1,3,6,7^ A key insight that is usually missing from these data, however, is whether the use of fentanyl with other substances was intentional or unintentional. Systematic data on fentanyl contamination of the illicit drug supply in the U.S. is lacking.^8^ Unintentional use of fentanyl through contamination of the drug supply is believed to be a major contributor to the surge in overdoses.^9^

In the absence of definitive information regarding the content of street drugs, several community-based studies have explored *perceived* unintentional fentanyl use among people who use drugs (PWUD) in diverse localities such as Philadelphia,^10^ New England,^11,12^ Baltimore,^13^ Los Angeles and San Francisco.^14^ This approach acknowledges that, based on their experience, PWUD often have a deep understanding of illicit drug composition and effects. For example, a study of 60 PWUD in Dayton, Ohio found that 84% of participants who reported perceived use were positive for fentanyl in toxicology.^15^ By contrast, a Maryland study found low concordance between perceived fentanyl use and fentanyl positive urinalysis,^16^ and a Massachusetts study had mixed results in terms of the accuracy of perceived fentanyl use.^17^ It is fair to say that fentanyl contamination has increased the difficulties associated with predictable, consistent and safe substance use in the context of illicit drug markets.

In an ongoing study regarding polysubstance use among people who use opioids (NIDA grant #R01DA049761), we found that nearly a third of participants who did not endorse fentanyl use by self-report tested positive when screened using point-of-care urinalysis. This paper describes the characteristics, reactions and experiences of participants who were informed they had consumed fentanyl unintentionally. We also describe how our research team decided on appropriate disclosure and harm reduction counseling procedures. Our findings contribute to research regarding unintentional fentanyl use among PWUD in three ways. First, because we conducted urinalysis, fentanyl exposure was verified rather than ‘perceived’. Second, we explored the reactions of PWUD immediately upon learning they were unwittingly exposed to fentanyl, as well as the reflections of a subset of participants several weeks later. Third, we discuss the impact of the discordance between self-report and UA results as an unanticipated event for the research team, as well as the people participating in the study.

## Methods

Our findings are drawn from an ongoing, community-based study of polysubstance use and overdose risk in Oakland, CA. Eligibility criteria include the use of an opioid, plus the use of alcohol, benzodiazepines, cocaine, methamphetamine, or a second type of opioid in the past 3 days. The screening procedure begins with a checklist of specific substances (e.g., heroin, crack, fentanyl etc), which participants respond ‘yes’ or ‘no’ to having used in the past 3 days. If participants endorse use of an opioid plus another qualifying substance in the past 3 days, they are then asked to provide a sample for urinalysis (UA). For convenience at the community field site, we use a rapid point-of-care collection test rather than sending samples out for laboratory testing. We use the 13 Panel T-Cup® Drug Test Cup. According to information provided by the manufacturer, the specificity for fentanyl detection with this test is 100% (CI: 84.5%−100%). Study procedures were approved by the IRB at RTI International, and informed consent was obtained from all participants.

Because they were intended as confirmation of what participants told us by self-report, we did not plan to communicate UA results them with participants, nor to record them as data. However, in week 4 of the study, a participant screened positive for fentanyl without having endorsed its use by self-report. Study staff recognized the potential risk of unintentional fentanyl use to the participant’s health and contacted the Principal Investigator. Upon discussion, we made the decision to inform the participant that they had screened positive for fentanyl. In procedures that were later formalized, we told the participant that their UA tested positive for fentanyl, and while the rapid UA test was not always 100% accurate, it was likely they had unknowingly used fentanyl in the past few days. We then provided a supportive space for the participant to reflect on the discordance we found. At the end of the discussion, study staff counseled them regarding harm reduction strategies for potentially contaminated substances, such as using fentanyl test strips and ‘tasting’ substances before using a full dose to gauge their strength. Fentanyl test strips and nasal naloxone are routinely offered to all participants at the study site.

Given the recurrence of this event, we developed a sub-study to investigate the unforeseen outcome of discordant information regarding fentanyl use. We reviewed the study staff’s contemporaneous observational notes about each interview, which included reflections on conversations when participants learned that their UA was positive for fentanyl. In addition, we conducted follow-up qualitative interviews with a convenience sample of participants (n = 6) 4–6 weeks after we informed them of their positive UA results. We conducted a thematic analysis of notes and transcribed interviews. In addition, we incorporated quantitative data from the study’s baseline survey to describe participant characteristics, substances use and overdose experiences.

## Results

As of this writing (4/1/2023), there were 95 people enrolled in the source study. Among the 58 participants who did not endorse fentanyl use, 17 (29%) tested positive in UA. All confirmed that they had not intentionally used fentanyl use in the past 3 days (as they reported in the screening process); furthermore, baseline data show that none reported fentanyl use in the last 30 days. Nearly all (n = 15) reported heroin use in the past 30 days, and most reported marijuana and alcohol use (Table 1). The two participants who did not report heroin use had used benzodiazepines and opioid pills purchased on the street, in addition to methamphetamine.

The participants who experienced unintentional fentanyl use were differed from the overall sample in a few ways. More identified as African American or Black (94% vs 67%, p = .02) and more used heroin (88% vs 63%, p = .04). In addition, a higher proportion reported being unhoused at some point in the past year (55% vs. 24%, p = .02).

### Participant Reactions

Overwhelmingly, participants appreciated learning about their fentanyl-positive UA result. They felt this was important information for managing their overdose risk. As recorded in interviewer notes,

…she was grateful of the awareness that her drugs were being tainted with fentanyl so she could use smarter.

He was very surprised/concerned that he tested positive for fentanyl and was deeply grateful that we let him know.

Most participants expressed anger, surprise or discomfort at learning they may have unintentionally used fentanyl, driven primarily by knowledge regarding the potency of fentanyl and fear of accidental overdose. As one participant said,

I was a little bothered by the fact that I tested positive for fentanyl because I don’t mess with that, I’m like, I’m actually rather afraid of it

It was common for participants to recall friends or acquaintances who had overdosed on fentanyl, and participants told stories of losing friends or ‘bringing people back’ from overdose with naloxone. One woman expressed the sense of personal vulnerability unintentional fentanyl use evoked:

I would just hate for something to happen and my son to have to find me. I’ve had a lot of thoughts about my mortality lately and that’s just one more thing for me to have to stress off of.

One participant informed us that he was not surprised by his result, because a friend had tested positive for fentanyl at a treatment program, and they figured out it was in some methamphetamine they had used together. Another had tested positive for fentanyl at his doctor’s office the week prior to our study, an event which had initiated a discussion with his doctor about starting treatment with buprenorphine.

### Retrospective Sense-making

When processing the information that they had unintentionally used fentanyl, it was common for participants to think back to a recent drug use episode that felt unusual. Among those who typically use heroin, they recalled episodes in which the high or the “nod” was stronger than usual. “It was just too much of a good high,” said one participant. Retrospectively, they attributed their experience to the presence of fentanyl, as noted by an interviewer:

According to her urinalysis, she tested positive for fentanyl, so I informed her. As many others, she was surprised, and said that that likely explains why she has been feeling so good and catching a nice nod on this batch

Despite describing the experience as better than usual in some ways, it was rare for participants to say they wanted to start using fentanyl intentionally. Most felt it was too risky. One participant did not want to be burdened with an additional addiction:

You’re already doing a downer, which is the heroin, and then you turn around and you put some fentanyl in you, too, that’s double downer because now you have two habits, you know.

Another participant said he had tried fentanyl intentionally in the past and strongly disliked the experience. In his words, “It was like being dead.” He reported being ‘out of it’ for hours and grateful that he had been around friends who he could trust to protect him.

### Risk Reduction

In terms of reducing the risk of using contaminated substances, people talked most often about protecting themselves by being careful regarding drug sources. A common response upon learning about unintentional fentanyl use was to try to recall who they purchased potentially contaminated drugs from:

Now we’re backtracking, so who did we buy this from, you know what I’m saying, and you’re trying to like go back and trying to think about who did you get the dope from and make sure we don’t buy dope from that dude again.

Some participants focused on times they did not purchase drugs from their regular dealer, or times when they were given drugs by someone they used with. As one participant said, “it’s like now I got to really screen who I mess with.” Others pointed out that sometimes they have to buy drugs from an untested source, and sometimes dealers might lie about the content to make a sale.

Others talked about using smaller doses of drugs at a time, in case they were contaminated. None of the participants reported ever using fentanyl test strips to check their drugs, even though they are provided by local harm reduction programs. Skepticism was voiced about the practicality of using them: “if you’re heroin sick you’re not going to take the time to test it and see if it got fentanyl in it.” After learning of unintentional exposure, some participants took naloxone from study staff, and only a few took the fentanyl test strips that were offered.

Many participants expressed some level of resignation about being unable to avoid fentanyl contaminated drugs altogether.

You can’t avoid it, you know, because I might go buy a piece of dope tomorrow and it could have some in it, you know. But the sad thing is, it might have too much in it… That’s part of the risk now (7012)

In fact, as the study has continued, we have found participants endorsing fentanyl use based on the working assumption that one or more drugs they used was likely contaminated. We have since clarified that we are asking them to report intentional fentanyl use only.

## Discussion

People who use opioids with other substances have borne the brunt of the third and fourth waves of the overdose epidemic.^18,19^ Concern about fentanyl-related overdose is widespread.^20^ Social and structural factors that create the substance use risk environment, such as poverty, unpredictable drug supply and the criminalization of addiction, shape the options and actions of people trying to stay alive and safe while using drugs. Some of the actions described in this paper, such as only buying drugs from trusted dealers, seem fairly common and have been mentioned by PWUD in several communities.^15,20–22^ Other research describes how PWUD examine substances for physical characteristics of fentanyl contamination.^12,23^ In response to the information that they had inadvertently used fentanyl, it was common for participants in our study to recount recent drug use experiences that felt atypical, usually involving a stronger than expected level of intoxication. Their stories concur with studies of perceived fentanyl use, in which participants described their physical responses as a way of deducing that they had used fentanyl.^12,23^

Study participants were grateful that the research team shared the information that their UA results were positive for fentanyl. All were aware of the growing presence of fentanyl in the drug supply -- indeed some felt exposure was inevitable – but the personal experience of an unanticipated positive test heightened awareness of risk. This suggests a potentially useful role for medical clinics, methadone programs and other entities that conduct UA. By providing results in a supportive manner, they can equip people with a means to compare their intended substance use with UA results and act on the information as they wish. For example, one participant was motivated to discuss buprenorphine treatment with their provider. Currently, fentanyl test strips are provided by many harm reduction programs as a means of testing substances before they are used. Partly due to issues like not wanting to waste drugs and not wanting to wait before using drugs, uptake is uneven.^10,24^ Harm reduction providers also caution about the ‘chocolate chip cookie effect,’ where the test strip may not encounter the portion of drug that is contaminated. This could produce a false negative result and, dangerously, a false sense of assurance. An additional harm reduction option could be for PWUD to use fentanyl test strips as originally intended – as a rapid UA test – after substance use, particularly when they have an atypical experience that may indicate contamination. Opportunities to confirm these experiences with UA test results could provide potentially useful information to inform future purchases and decisions about how much to use.

It is notable that the study team did not consider the complications fentanyl contamination might create when we initiated our routine screening process. We did not plan for the contingency of discordant results. Our hope is that this may be a useful lesson for other substance use researchers. Upon finding our first case of discordance between self-reported substance use and UA results, we had to weigh several factors. We considered that perhaps participants omitted fentanyl in self-report data, but saw no incentive to do this, since the eligibility criteria for the study was polysubstance use and people reported use of many other substances. We reflected on formative work conducted early in the study, which suggested that fentanyl had a poor reputation and appeared to be less accepted in Oakland than across the bay in San Francisco. A countervailing consideration, however, was the small possibility that the UA results were inaccurate, since we weren’t conducting ‘gold standard’ laboratory testing; this is the reason we had not included provision of UA results in the original study protocol. Balancing all these considerations, we concluded we should inform participants about the results, a decision supported by our IRB. This information was received gratefully participants, and a routine research procedure became a useful harm reduction measure we shared with participants.

There are several limitations to this study. It is possible that stigma led participants to misreport their fentanyl use, even though the study created no incentive to do so. There is a small possibility of false positive UA results, particularly if any participants were taking antipsychotic medication.^25^ The sample for the study is small and limited to one geographical location. In addition, this study was initiated in response to an unanticipated research event, so we were not testing any pre-existing hypotheses. That said, we believe we were uniquely positioned to examine the experience of learning about unintentional fentanyl use among people who use multiple substances.

PWUD all over the country are struggling to manage the vagaries of illicit drug markets and reduce their risk of overdose. In addition to strong and ongoing support for harm reduction interventions, there is a need for broad and routine monitoring of the illicit drug supply. Given the local nature of drug markets, local health departments might be best positioned to regularly monitor illicit drug content and share these data. One possible model is the California Overdose Surveillance Dashboard, which provides Statewide and County-level data that is updated monthly (https://skylab.cdph.ca.gov/ODdash/?tab=Home). The provision of additional drug supply data through a resource like this could be make life-saving information accessible to PWUD, harm reduction providers and other affected community members.

## Figures and Tables

**Table 1:** Selected characteristics of participants with unintentional fentanyl use (N = 17)

	n	%
Age - *mean, median (range)*	54.5, 56.0	(28–67)

Female	10	59
Male	7	41

Race		

Black or African American	16	94

Other	1	6

Substance use past 30 days		
Alcohol	12	71

Marijuana	14	82

Crack cocaine	9	53

Powder cocaine (by itself)	7	41

Heroin (by itself)	15	88

Speedball	7	41

Methamphetamine	7	41

Methadone (street)	1	6

Benzodiazepines (street)	5	29

Opioid pills (street)	3	18

In methadone program	5	29
Ever overdosed	5	29
Overdosed past year	1	6
